# Monolayer formed by l-Asp-based gemini surfactants self-assembled in 1D nanostructures†

**DOI:** 10.1039/c9ra06390k

**Published:** 2019-10-16

**Authors:** Borislav A. Anchev, Daniela S. Tsekova, Kristina M. Mircheva, Nikolay A. Grozev

**Affiliations:** Department of Organic Chemistry, University of Chemical Technology and Metallurgy St. Kliment Ohridski Boulevard 1756 Sofia Bulgaria d_tsekova@abv.bg; Department of Physical Chemistry, Faculty of Chemistry and Pharmacy, Sofia University St. Kliment Ohridski, 1 James Bourchier Boulevard Sofia 1164 Bulgaria

## Abstract

Herein, studies on the surface activities of newly synthesized l-Asp-based gemini surfactants, both nonionic and anionic, are presented. Conductometry, tensiometry, and the Langmuir–Blodgett (LB) film technique were applied for this purpose. *π*–*A* isotherms were obtained with a Langmuir trough and Wilhelmy balance. The structures of the monolayers assembled at the air/water interface and those deposited as LB films were studied *via* Brewster angle microscopy (BAM) and atomic force microscopy (AFM). The 2D films formed by the anion-active compounds show a well-known pattern of a monolayer film, whereas the nonionogenic amphiphiles have been found to be 1D structures with nano-widths and micro-lengths that align with each other during the process of compression; this is the first study where the organization of 1D fibrils in 2D films during compression is reported. The scanning electron microscopy (SEM) study reveals that 1D nanostructure formation is an intrinsic tendency of these molecules as not only nonionogenic surfactants, but also the anion active representatives have been constructed in the solid state by fibrillary structures.

## Abstract 2

l-Asp-based gemini surfactants form primary fibrils (1D structure) that interact with each other and arrange in a film (2D structure).

## Graphical abstract

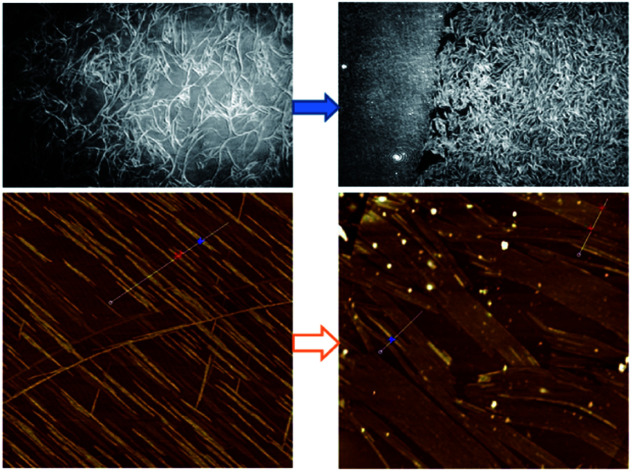

## Introduction

**Fig. 1:**
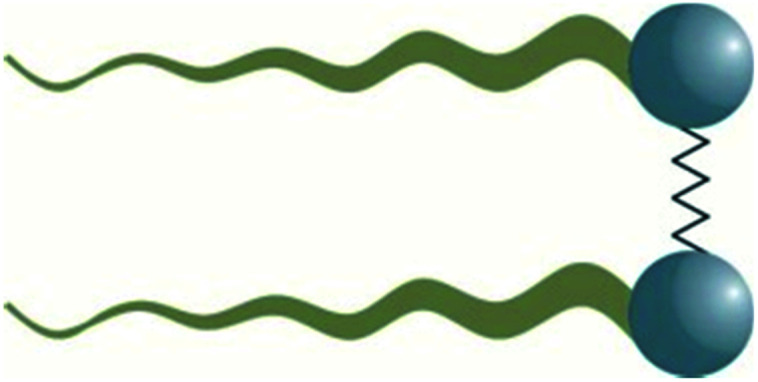
Molecular structure of gemini surfactants. The two hydrophobic tails are presented as long chains. Both hydrophilic heads are presented as circles, and the spacer between heads is depicted as a short wavy line.

Gemini surfactants are relatively new-generation surfactants.^1,2^ A typical gemini surfactant molecule (Fig. 1) consists of two hydrophobic tails and two hydrophilic heads connected *via* a flexible spacer. This construction of the molecule is responsible for the well-known unusual physicochemical properties of these surfactants;^3^ data obtained from different studies demonstrate that these molecules have better surface-active properties, such as more effective lowering of the surface tension and reduced critical aggregation concentration (CAC) by at least one order of magnitude, than the monomer analogues with the same chain length.^1–4^ Nowadays, in addition to the requirements of high surface activity and chemical stability, there is an increasing demand for biocompatibility, biodegradability and non-toxicity in the field of surfactants.^5,6^ For this reason, new surfactants on the basis of natural sources have been designed. Moreover, the introduction of amino acids, which are building blocks in living organisms, as building blocks for gemini surfactants aims to fulfil the modern requirements of eco-friendly, high-surface active compounds.^7^ In recent years, numerous studies have been reported on amino acid-based amphiphiles, revealing their remarkable properties in the bulk or at the air/water interface;^7–10^ one of the main findings for gemini natural amino acid-based surfactants (similar to general gemini surfactants) is that they have adsorption properties *e.g.* cmc values well below those of the monomeric constituents. Silva *et al.*^11,12^ reported the synthesis of a series of l-Ser-based gemini surfactants, which possessed cmc values lower than those of the conventional gemini bis-quats. In other studies,^8,10,13–15^ authors have described the synthesis and investigation of the surface activities of l-Arg-based gemini amphiphiles. Moreover, gemini surfactants based on l-Lys have been well studied and reported.^16,17^ However, limited studies have been reported on surfactants containing acidic amino acids,^18,19^ and no study has been reported on the gemini structures of these surfactants. Moreover, only few studies have been performed on amino acid-containing surfactants under the conditions of the formation of a Langmuir thin film. Rogalska *et al.* presented data for the monolayers formed by gemini amphiphiles based on the nonpolar amino acids Ala, Val, Leu, and Phe.^20,21^

Herein, we report eight new compounds, derivatives of l-Asp, that structurally belong to the group of gemini surfactants (Fig. 1). Their molecules include l-Asp as the hydrophilic part and the aliphatic acid acyl residue as the hydrophobic part. These compounds have been divided into two structurally related series: four of the compounds contain two free carboxylic groups (compounds 2a–d) and the other four are their double benzyl esters (compounds 1a–d); moreover, studies on their surface properties, such as critical aggregation concentration (CAC) and surface tension value at CAC (*σ*_CAC_), are presented. The effects of the changes in the behaviour of the amphiphilic molecules studied herein on the water/air interface during compression were examined by determining the surface pressure–area per molecule (*π*–*A*) isotherm. Morphological observation of the compression process at the air/water interface was carried out by Brewster angle microscopy (BAM), and the related images were obtained; some of the layers were transformed into Langmuir–Blodgett (LB) films onto a mica surface and monitored by AFM; the results obtained show that the interactions of the molecules of nonionogenic surfactant lead to the formation of two types of structures: primarily, the formation of fibrils (1-dimensional/1D structure), and secondarily, the formation of a film (2-dimensional/2D structure) *via* the interaction of fibrils.

## Results and discussions

**Scheme 1:**
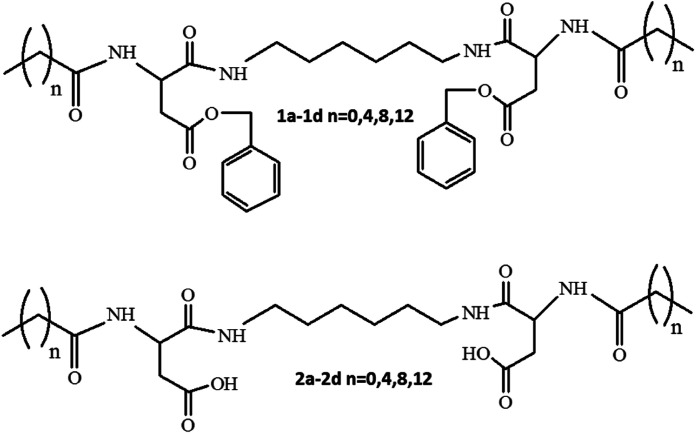


Herein, the compounds 1a–d and 2a–d (Scheme 1) were synthesized according the schemes presented in the ESI.† The length of the aliphatic tails was modified to study its influence on the surface properties and supramolecular structures of this type of molecules; the derivatives containing an acetyl residue (1a and 2a) did not exhibit surface properties. They were synthesized to compare the physicochemical properties of individual series members. Real surface activity was determined for the other six derivatives, three of which were nonionogenic (1b, 1c, and 1d) surfactants, whereas the other three were anion-active (2b, 2c, and 2d) surfactants, whose properties were also related to the availability of either esterified or free carboxylic groups.

### Monolayer formation

**Fig. 2:**
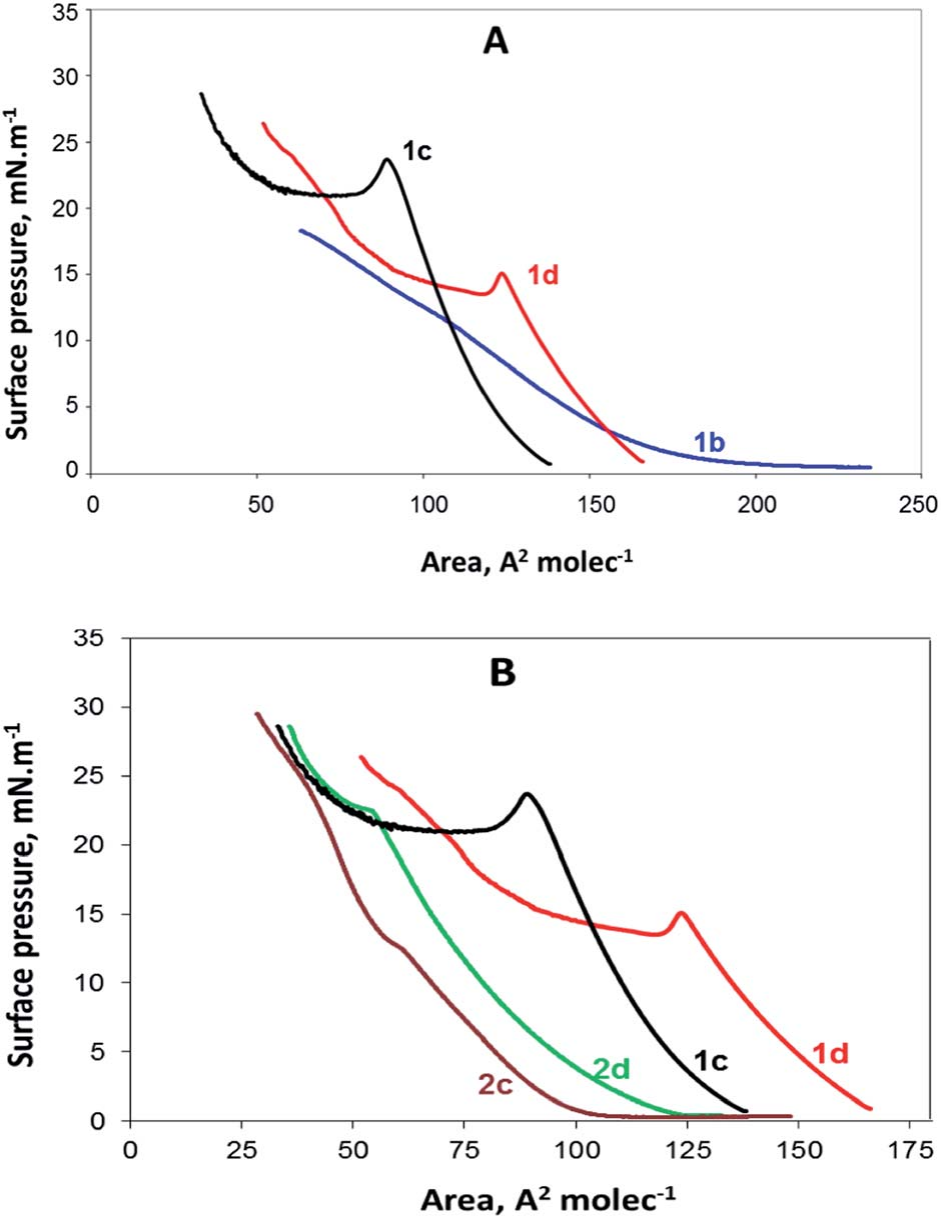
(A) *π*–*A* isotherms of the gemini surfactants 1b, 1c and 1d containing benzyl ester groups and (B) *π*–*A* isotherms of all compounds (1c, 1d, 2c, and 2d) having *n* = 8 and *n* = 12.

Newly synthesized compounds that formed stable films on the water surface were investigated using a Langmuir trough. The results obtained in the form of surface pressure–apparent molecular area (*π*–*A*) isotherms are shown in Fig. 2.

In Fig. 2A, the *π*–*A* isotherms of the gemini surfactants containing benzyl ester groups are presented. They are presented together to compare the behaviour of the monolayers of 1b, 1c and 1d. As observed, no collapsing point was detected for the compound 1b. According to the isotherm, at the beginning, *π* is close to zero, and initial compressing induces the rearrangement of the molecules at the air/water interface; however, further pressure leads to the parallel condensation and dissolution of the compound instead of a coalescence. The other two isotherms presented in Fig. 2A are similar to each other and show that the compounds 1c and 1d undergo collapse, which is typical for surfactant monolayers.

The compound 2b contains the same alkyl tails as 1b; however, its hydrophilic part includes two free carboxylic groups instead of the ester benzyl groups of 1b. Thus, 2b is expected to be more soluble than the compound 1b. Indeed, the *π*–*A* isotherm of 2b resembles the *π*–*A* isotherm of 1b with missing collapse, and because of this, it is not presented herein.

The *π*–*A* isotherms of the compounds 2c and 2d are presented in Fig. 2B. Moreover, the isotherms of the compounds 1c and 1d are included in the same figure for comparison with those of the compounds 2c and 2d. All these monolayers are in a liquid extended (LE) state up to the collapse point, determined by the values of compressibility modulus;^22^ as well-observed from this figure, the derivatives of decanoic and tetradecanoic acids both with free and esterified carboxylic groups have provided *π*–*A* isotherms typical for fatty acid and surfactant structures.^23^ It seems that the isotherms and the compressibility modulus of our compounds are similar to the data reported for other amino acid-based amphiphiles.^20,21^

The isotherms of the compounds 1b and 1c and those of the compounds 2c and 2d are obviously different; this is most probably due to the nature of the head groups and subsequent head group–subphase interactions.^24^ Obviously, compounds with esterified carboxylic groups (1c and 1d) and those with charged carboxylate groups (2c and 2d) take part in interactions of different types and strength. The existence of two benzyl groups linked to the head makes the head of the compounds 1c and 1d much larger than that in the case of the compounds containing free carboxylate groups. Some of our preliminary studies suggest that the benzene rings are not anchored perpendicular (not in the upright position) in the water subphase but are rather lying sideways.

The data obtained by compressing a monolayer can be used for the mean molecular area (MMA) determination, also defined as the average amount of space explored by one molecule. Obviously, at the moment of collapse, we can find the lowest MMA as after this, they form a new phase and enter inside the water subphase. The MMAs reported herein represent the data at the point of collapse.

In Fig. 2B, it is clear that the area occupied by the molecules with benzyl groups is significantly broader than that occupied by the molecules with free carboxylic groups. The MMA for the compound 1d is 125 A^2^, whereas that for the compound 2d is 60 A^2^. Similarly, the MMA for the compound 1c is 95 A^2^ and that for the compound 2c is 65 A^2^. Although the differences in MMA for the both pairs of molecules (1d & 2d and 1c & 2c) is expected to be equal area, corresponding to two benzyl groups, we found from the *π*–*A* isotherms two different values: 55 A^2^ and 30 A^2^, respectively. The most probable explanation herein is that the MMAs determined by the *π*–*A* isotherms have some deviations from the real values due to the interactions between surfactant molecules, leading to self-organised polymolecular structures.

**Fig. 3:**
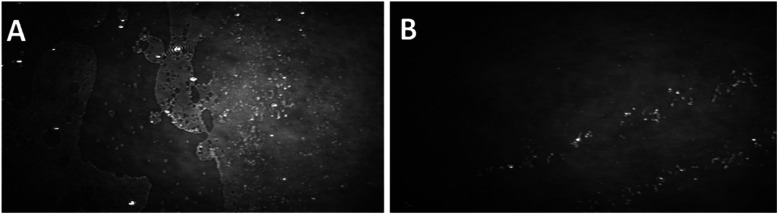
BAM images (sizes 720 × 400 micrometres) of a monolayer formed by 1b. (A) At the beginning of compressing, *π* = 2 mN m^−1^; (B) at *π* = 18 mN m^−1^.

**Fig. 4:**
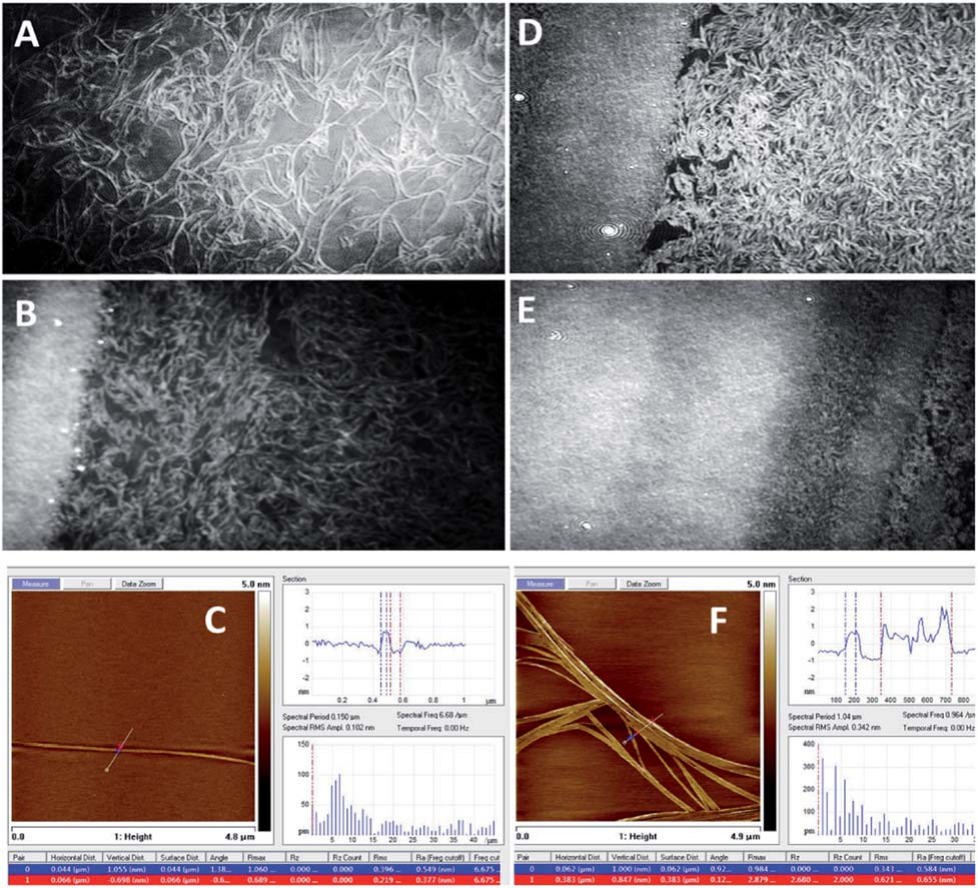
BAM images (sizes 720 × 400 micrometres) of the monolayer of the compound 1c (A, B, D, and E) and representative atomic force microscopy (AFM) images of the samples of the compound 1c coated on mica substrates (C and F). Before the collapse (A–C), (A) *π* = 15 mN m^−1^; (B) *π* = 22 mN m^−1^; and (C) *π* = 15 mN m^−1^; after the collapse (D–F), (D) *π* = 22 mN m^−1^; (E) *π* = 26 mN m^−1^; and (F) *π* = 22 mN m^−1^ (see ESI Fig. ESI-4† for the enlarged AFM images of C and F).

**Fig. 5:**
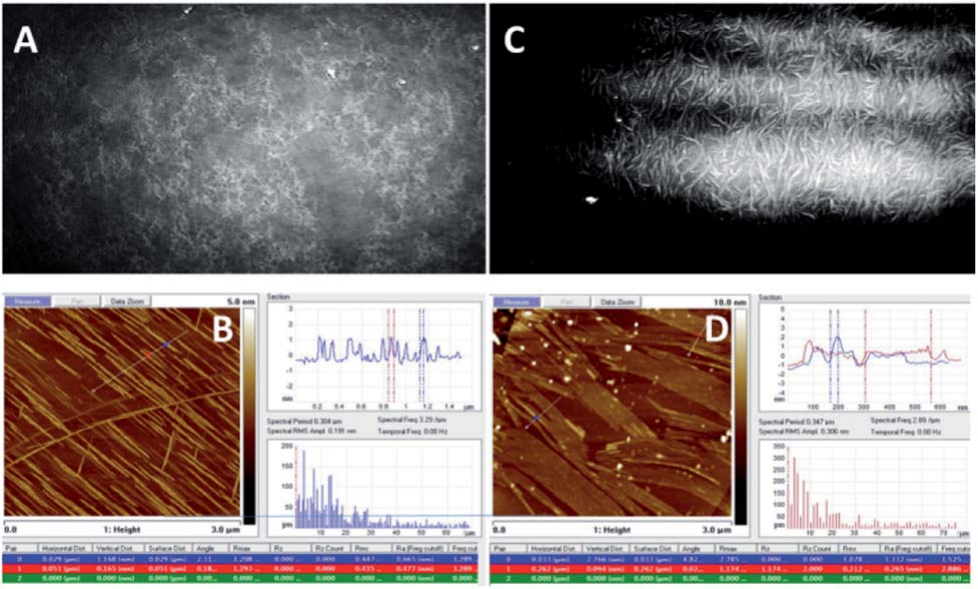
BAM (sizes 720 × 400 micrometres) and AFM images of the compound 1d. (A) (BAM) and (B) (AFM): before the collapse, at *π* = 13 mN m^−1^; (C) (BAM) and (D) (AFM): after the collapse, at *π* = 27 mN m^−1^ (see ESI, Fig. ESI-5† for the enlarged AFM images of B and D).

To gain insights into the structure of the monolayer and prove the existence of aggregates therein, microscopic techniques were used. Fig. 3–5 show the BAM images (720 × 400 micrometres) of the monolayers obtained from the compounds 1b, 1c and 1d. In the monolayer formed by 1b, the initial aggregation of the compound at the air–water interface most probably occurs during the evaporation of the solvent from the thin solution layer. At the beginning of compression, at 2 mN m^−1^, the 2D layer cracked into small islands, and scattered spherical aggregates with diameter less than 10 microns were observed (Fig. 3A). When the barrier was moved, these small islands coalesced, forming larger islands, until a uniform film was built. Fig. 3B presents an image when the barrier is moved until *π* = 18 mN m^−1^ (exactly before the compression is stopped); herein, a uniform film and spherical aggregates arranged in strips are visible.

Fig. 4 shows the BAM images of a monolayer formed by 1c. The layer formation after solvent evaporation was monitored at different steps of moving the barrier. Surprisingly, very well-shaped twisted filaments with the length of several hundred microns and thickness of several microns were observed herein. Due to the shifting of the barrier, the fibres became closer and entangled with each other; thus, the structure of the film became significantly compact to collapse, and the densest packaging was observed.

The images of the compound 1d are presented in Fig. 5, where it is visible that the 1d layer is constructed by filaments that are comparable to the dimensions of 1c; moreover, during the compression of 1d, a similar behaviour to 1c was observed. Herein, the impressive feature is that upon compression, the filaments gather together and align with each other along their axes (Fig. 5B). Eventually, they pile up upon one another.

**Fig. 6:**
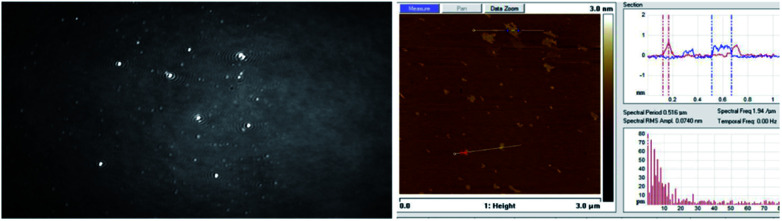
BAM (sizes 720 × 400 micrometres) and AFM images of the compound 2d (see ESI, Fig. ESI-6,† enlarged AFM image).

The BAM and AFM images of the monolayers formed by 2c and 2d show only some globular structures, as presented in Fig. 6.

**Fig. 7:**
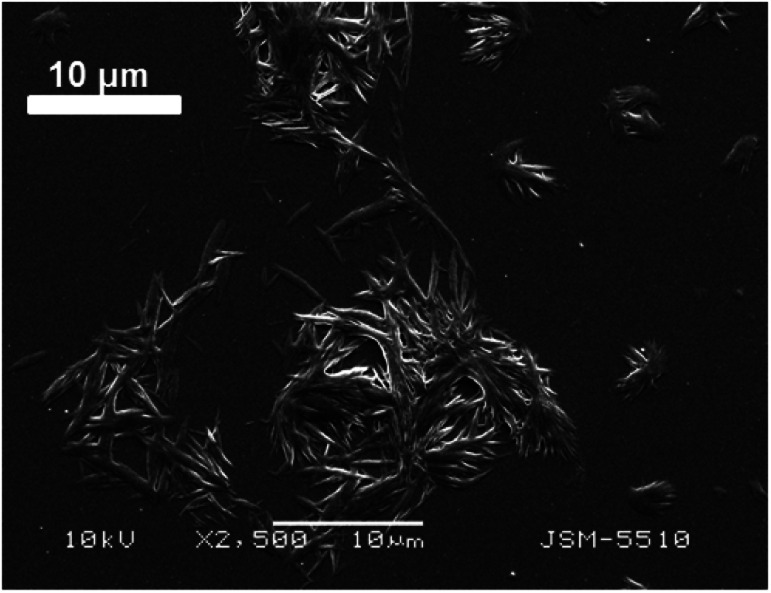
SEM image of the compound 1d on a glass surface obtained from a chloroform solution after the evaporation of chloroform.

For comparison, as an analogue of the thin solution layer on the water subphase, a droplet of the chloroform solution of the compound 1d was put on a glass surface, the solvent was allowed to evaporate, and then, SEM was performed for observation. It is visible from Fig. 7 that the solid obtained possesses a tendency for fibre formation, but herein, the fibres are short and almost straight, whereas the fibres in the monolayer are significantly longer and curvy. From these two experiments, we can conclude that the morphology of the fibres and their relationship with one another depend very much on the sub-phase on which they are formed.

**Fig. 8:**
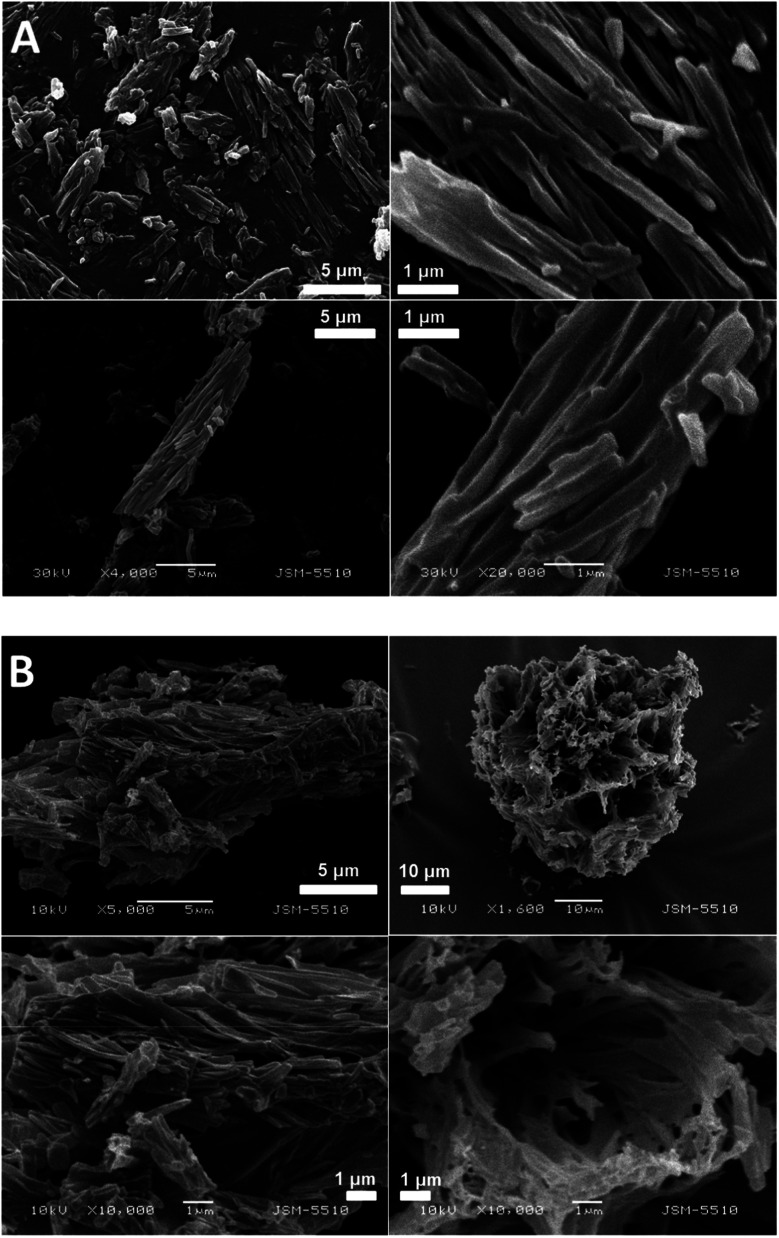
SEM images of the solid pieces of 1c (A) and 1d (B).

**Fig. 9:**
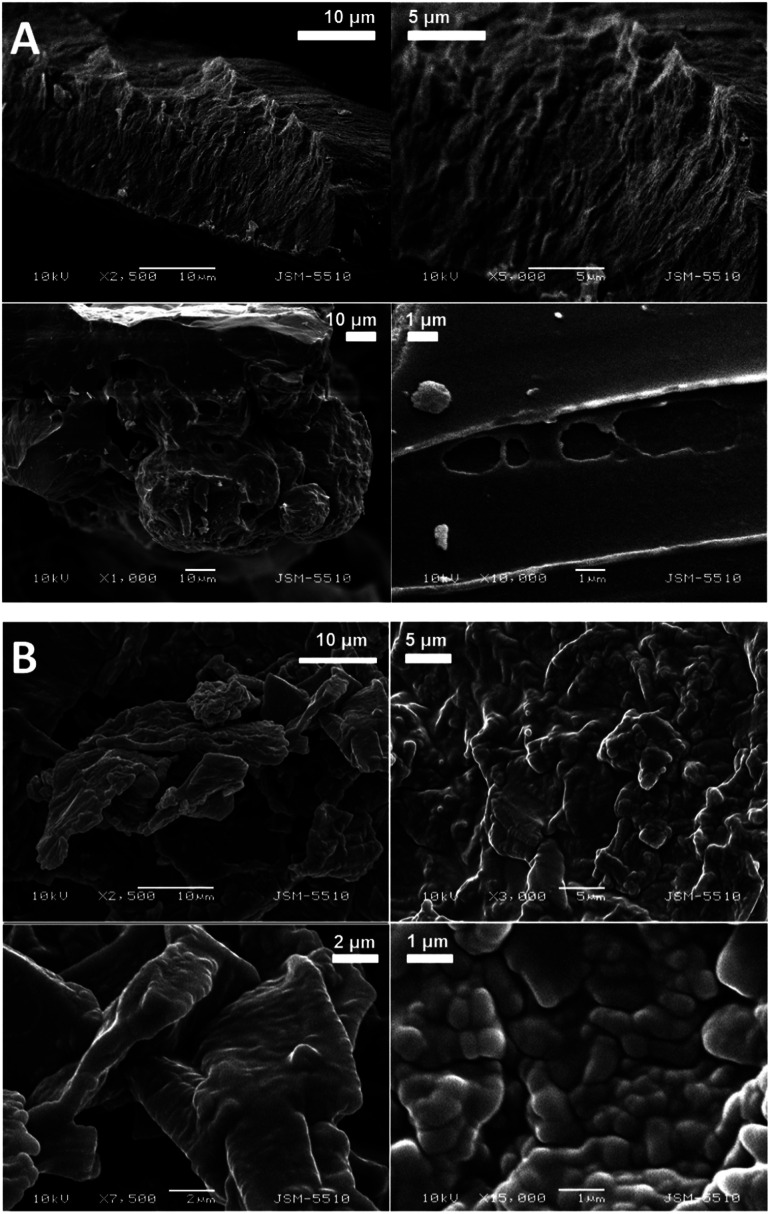
SEM images of the solid pieces of 2c (A) and 2d (B).

**Fig. 10:**
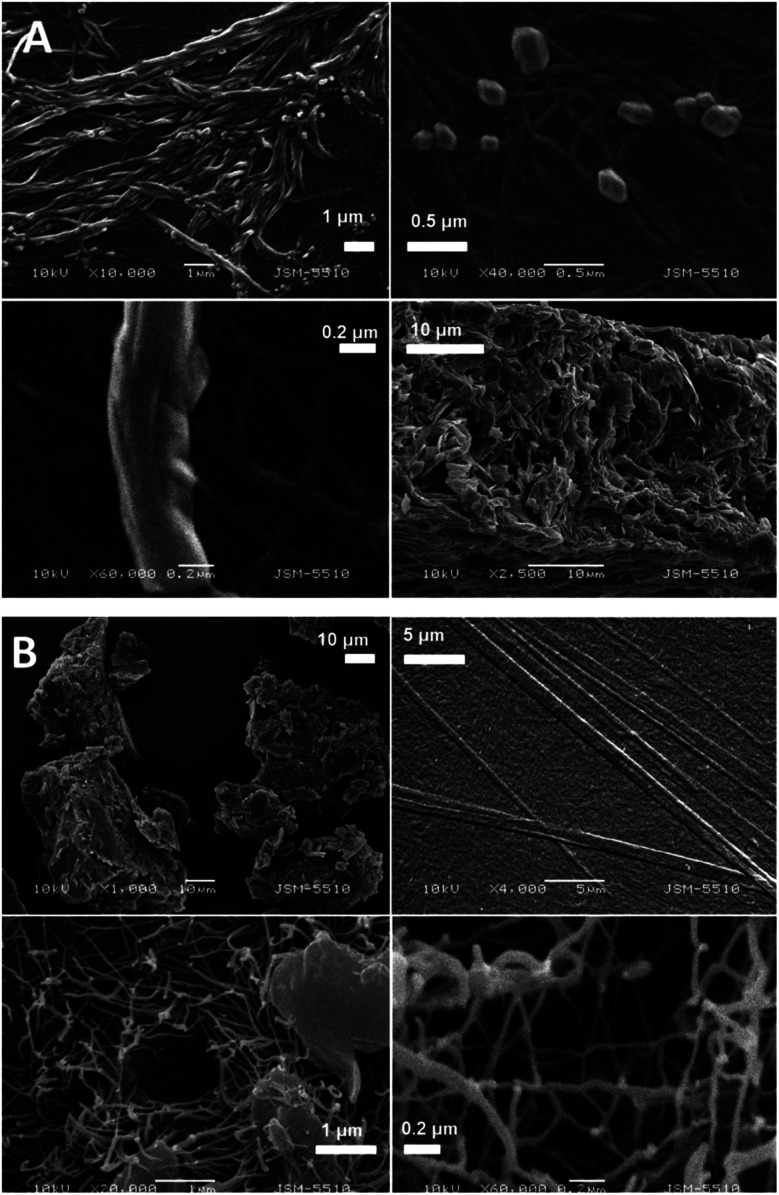
SEM images of the solids obtained from the sodium salts of the compounds 2c (A) and 2d (B).

The SEM images of the samples show that there are some variations in their micro- and nanostructures based on the way they have been prepared; by comparing the bulk structures of the compounds 1c and 1d (Fig. 8), 2c and 2d (Fig. 9) and the sodium salts of 2c and 2d (Fig. 10), we can see that in all the cases, the structures contain very thin filaments adhering in different ways to each other and forming porous structures of pieces.

The compounds obtained from water solutions have different morphologies in the bulk (inside the piece) and on the surface. Most probably, in solution at high supersaturation, the solute condenses as a residue in solid pieces. Before drying these pieces, they were wetted and covered by a saturated solution. The evaporation of water led to the deposition of the dissolved molecules as a thin layer around the pieces, typical for surfactants. Because of this, in the images obtained by SEM, two patterns are well visible: structured bulk (fibrillose, porous, *etc.*) and a smooth surface.

In our previous study, we have found the fibrillar nanostructure formation by the sublimation of bolaamphiphilic l-valine derivatives.^25^ Obviously, many low-molecular-weight organic molecules, derivatives of alfa amino acids, manifest the tendency to form 1D assemblies through intermolecular interactions, which is an intrinsic property of these molecules.

The formation of nanostructures with similar dimensions by inorganic substances has been reported by other authors.^26^

### Critical aggregation concentration (CAC)

**Fig. 11:**
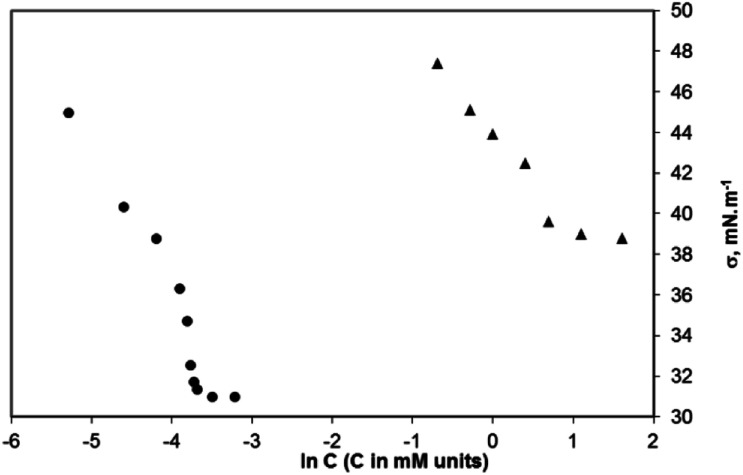
Surface tension *vs.* logarithm of concentration curves for the sodium salts of 2c and d. Filled circles – double sodium salt of 2d, CAC = 0.025 mM; filled triangles – double sodium salt of 2c, and CAC = 2 mM; for the double sodium salt of 2b – CAC was found to be above 20 mM. All measurements were conducted at 22 °C.

The minimum concentration at which the surfactants in the bulk aqueous solution start forming aggregates is the critical aggregation concentration (CAC). The aggregation process for the surfactants synthesized by us was studied conductometrically and tensiometrically by measuring the solutions with concentrations above and below the CAC. The surface tension *vs.* ln *C* plots for two of the studied surfactants (sodium salts of 2c and 2d) are presented in Fig. 11. The CAC values determined conductometrically and tensiometrically are summarized in Table 1. Data for the CAC of the three compounds obtained conductometrically (see ESI, Fig. ESI-2A–C†) are close to those found tensiometrically (Table 1). The sodium salt of compound 2b has a CAC = 10.0 mM, conductometrically estimated (tensiometrically, it has been found to be 20.0 mM); the sodium salt of the compound 2c has a CAC = 1.0 mM, conductometrically determined (2.0 mM – tensiometrically measured), and the sodium salt of the compound 2d has a CAC = 0.020 mM, conductometrically measured (0.025 mM tensiometrically measured).

**Table 1:** Results for CAC obtained by applying both tensiometric and conductometric methods

Compound	Number of C atoms in both tails	CAC (mM)	
		Conductometry	Tensiometry
2b – Na salt	12	10	20
2c – Na salt	20	1	2
2d – Na salt	28	0.020	0.025

Actually, the decrease in CAC with the increasing length of the aliphatic fragment in our experiments can be summarized as follows: a 10 times lower CAC has been found for 2c as compared to that for 2b, and a 50 times lower CAC has been found for the compound 2d as compared to that for 2c (see data in the Table 1).

CAC and the surface tension *σ*_CAC_ determined at the CAC are important parameters in describing the surface activity of amphiphiles. Table 2 provides a comparison of CAC and *σ*_CAC_ of the gemini surfactants synthesized by us and the others described in the literature (conventional and amino acid-based surfactants). In Table 2, we have presented the tensiometrically found CAC values.

**Table 2:** Comparison of critical aggregation concentrations (CAC) and surface tension at that CAC (*σ*_CAC_) of the gemini surfactants synthesized by us and literature data for others

	Compound	Type of surfactant	R^a^	CAC (mM)	*σ*_CAC_ (mN m^−1^)	Ref.
1	Sodium salt of 2b	Anionic	12	20	44.0	This paper
2	Sodium salt of 2c	Anionic	20	2	39.6	
3	Sodium salt of 2d	Anionic	28	0.025	31.4	
4	C_5_H_11_COONa	Anionic	6	730	Conduct	27
5	C_9_H_19_COONa	Anionic	10	74.5	32.6	28
6	C_13_H_27_COONa	Anionic	14	6.90	34.5	29
7	*N*,*N*′-Bisdodecyl-serine-aminopropane	Anionic	24	0.27	33.2	12
8	*N*,*N*′-Bisdodecyl-serine-aminohexane	Anionic	24	0.40	32.1	12
9	Sodium-1,2-bis-*N*-dioctyl-cystine	Anionic	24	0.001	40.8	30
10	Sodium didecaminecystine	Anionic	20	0.75	38.0	31
11	Sodium dilaurylaminecystine	Anionic	24	0.02	33.8	
12	Soidium-*N*-dilauroylsarcosinate	Anionic	24	0.95	33.6	32
13	Sodium-1,2-bis-*N*-dodecanoyl-β-alanine	Anionic	24	0.04	27.0	33
14	*N* ^α^, *N*^ω^-Bis (*N*^α^-lauroylarginine)α, ω-hexyldiamide dihydrochloride salts	Cationic	24	0.28	30.0	8
15	14-6-14	Cationic	28	5.62	42.5	34
16	12-6-12	Cationic	24	1.12	42.5	35
17	Sodium dodecyl sulphate SDS	Anionic	12	8.20	32.5	27

a R – number of carbon atoms in the aliphatic chain/s.

When the CMCs of the sodium salts of the fatty acids used for their synthesis were compared, *i.e.* caproic (row 4), capric (row 5) and myristic acid (row 6), the critical aggregation concentrations of the sodium salts of 2b–2d were found to have decreased 36 times (for compound 2b), 37 times (for compound 2c) and 276 times (for compound 2d), respectively, *i.e.* we achieved a higher surface activity at significantly lower concentrations.

With respect to the serine-based gemini surfactants listed in Table 2 (rows 7 and 8), the compounds obtained by us demonstrate a CAC that is one order of magnitude smaller at approximately the same *σ*_CAC_. For the cystine (rows 9–11), carcosine (*N*-methylglycine) (row 12) and β-Ala (row 13) anionic surfactants, CAC and *σ*_CAC_ values reported are close to those of the gemini surfactants obtained by us.

The results obtained for the compounds synthesised by us show comparable CAC and *σ*_CAC_ values to the data already reported by the other authors on gemini amino acid-based surfactants (see Table 2 and ref. therein). Only one compound, the derivative of β-alanine, provided better results in reducing the *σ*_CAC_.

When 2d was compared with the cationic gemini surfactants (rows 14–16) synthesized by us, the compound 2d again showed better values of CAC and *σ*_CAC_. For example, comparison with its conventional cationic analogue (14-6-14) bisQuats-bis-tetradecylhexamethylammoniumbromide (row 15) shows that 2d has a more than 200 times lower CAC and a significantly lower *σ*_CAC_.

The results obtained for the CAC and surface tension (*σ*) show that the new compounds have a well-expressed surface activity regardless of the propensity of the molecules to form filamentous aggregates.

## Experimental

### Synthetic procedures

Synthetic procedures are presented in detail in ESI.† They include chemical synthesis, purification and analysis of the product.

Chemical syntheses were performed in solution using TBTU as a condensing reagent and DIEA as a base.

Purification was achieved by recrystallization from appropriate solvent/s.

Analyses of the molecular structure and the purity of each of the new compounds were carried out by ^1^HNMR, ^13^CNMR, MS spectra, FTIR and melting point.

### Methods and apparatus

#### Monolayer deposition

The formation of the monolayers was achieved by the droplet deposition of a specified amount of the solutions over the available area of a Teflon trough (475 cm^2^). The subphase was either deionized water or an acidic water medium. The surface pressure *π* was measured using a KSV-2200 (Finland) surface balance equipped with a platinum plate.

#### Critical aggregation concentration (CAC) determination

The critical aggregation concentrations of the anionic surfactants were determined by applying a tensiometric and a conductometric approach. The solutions of known concentrations were progressively diluted and examined. The temperature was 25 °C. These measurements were performed in unbuffered aqueous solutions. The CAC value was determined by plotting the surface tension against the log of the concentration of an amino acid surfactant.

#### Apparatus used for the analysis of new compounds

^1^H and ^13^C spectra were obtained using the Bruker Avance-II ±600 MHz spectrometer. The ^1^H and ^13^C NMR chemical shifts are presented relative to TMS. Chemical shifts are expressed in ppm while the coupling constants are in Hz. The ESI/MS analyses were conducted using the Thermo Finnigan LCQ advantage ion trap mass spectrometer. The purity of the products and the progress of the reaction were checked by TLC on precoated plates of Silica gel 60 F254 (Merck). Spots on the TLC chromatograms were detected by the chlorine/*o*-tolidine reaction. The melting points were determined using the Kofler apparatus and are uncorrected.

#### Apparatus utilized for supramolecular structure study

Ramé-Hart model 290 automated goniometer with DROPimage Advanced v2.4 and WTW inoLab 720 with a conductivity cell Tetracon 325, BAM – Nanofilm_ultrabam (Accurion); Image sizes 720 × 400 micrometers. AFM – Nanoscope V (Veeco Instruments Inc.). Scale bare is indicated. SEM – Jeol scanning electron microscope JSM-5510 (Jeol Ltd.) was used for the observation of dried samples.

## Conclusions

Herein, new representatives of gemini surfactants were synthesized, whose molecules include l-Asp as a hydrophilic part and acyl residues of aliphatic acids as a hydrophobic part. They belong to two structurally related series: four of the new compounds contain two free carboxylic groups (compounds 2a–d) and the other four are their double benzyl esters (compounds 1a–d). Studies of the surface activities of these compounds show that the acetic acid derivatives (1a and 2a) do not possess surface activity. The other six are surfactants, three of which are anion-active (2b, 2c, and 2d) and the other three are nonionogenic (1b, 1c, and 1d). Tensiometric and conductometric measurements for the sodium salts of the anionic surfactants (2b, 2c, and 2d) showed really lower cac/cmc values than those of the conventional representatives with the same tail length (results are summarised in Table 2).

For example, comparison with the sodium salts of the parent fatty acids shows that the compound 2b has a 36 times lower CAC than caproic acid (C_5_H_11_COONa), whereas the compound 2c has a 37 times lower CAC than capric acid (C_9_H_19_COONa), and the compound 2d has a 276 times lower CAC than caproic acid (C_13_H_27_COONa); this means that the new surfactants achieve higher surface activity at significantly lower concentrations.

An increase in the length of the aliphatic fragment led to a 10 times lower CAC for 2c than that for 2b and a 50 times lower CAC for the compound 2d as compared to that for 2c (see data in Table 1).

Investigations on the formation of monolayers at the water/air interface show that the derivatives of decanoic and myristic acids in both anion-active and nonionogenic forms arrange into quasi-monomolecular films, providing typical surface pressure–apparent molecular area (*π*–*A*) isotherms. The compounds containing hexanoyl residues (1b and 2b) do not provide such an isotherm. The isotherms of the compounds in which the polar part is not charged and includes benzyl ester groups (1c and 1d) significantly differ from those of the compounds with charged carboxylate groups (2c and 2d). Moreover, two governing factors can be pointed: the area occupied by the head group at the air/water interface and the type of interactions with the subphase. The presence of two benzyl groups leads to a marked difference in the molecular area (MMA). All the isotherms are reproducible. The arrangements of the structure morphology during the compression of the monolayer were examined by BAM.

To the authors‘ best knowledge, this is the first time where the formation of fibres with nano- and microdimension ordering in a Langmuir monolayer has been observed by microscopic techniques (BAM and AFM).

Obviously, the molecules of the newly synthesized substances tend to self-organize into 1D supramolecular complexes. The fibres formed secondarily interact with each other under compression to form a dense 2D layer. The physical interactions responsible for formation and existing of this type monolayer is a matter to be explored. Since the isotherms of these compounds are reproducible and the isotherm-defined MMA values are constant (although they do not correspond to the actual size of a single molecule), we can conclude that the dimensions of the 1D aggregates under the same conditions are the same and have a construct behaviour, *i.e.* these aggregates can be deemed as polymeric molecules based on the physical bonds between monomers.

The pattern of fibre formation and their arrangement in a 2D film have been observed only for the compounds 1c and 1d, which belong to nonionogenic surfactants. Their molecules are double benzyl esters of the compounds 2c and 2d. On the other hand, the compounds 2c and 2d contain free carboxylic groups and represent anion-active gemini surfactants. The observation of the monolayers formed by 2c and 2d using BAM and AFM did not reveal a filamentous nature. Scanning electron microscopy images of the solid samples show that all six amphiphilic compounds 1b–1d and 2b–2d have a filamentous structure. Herein, some questions arise: how far the molecules with free carboxyl groups align in a 2D layer by forming thinner fibrils that are indistinguishable by techniques such as BAM and AFM, *e.g.* sodium salt of 2d forms filaments with a diameter of 20–30 nm (SEM Fig. 10B). To what extent the adhesion among the filamentous constructions can happen during compression, simple accumulation or for another reason to form a laminar structure by merging the boundaries between the individual threads? How stable is this type of monolayer (built by nanofilaments)? These and other questions require new studies on the observed phenomenon.

## Conflicts of interest

There are no conflicts to declare.

## Supplementary Material

RA-009-C9RA06390K-s001
