# Supplementary material for: Monolayer formed by l-Asp-based gemini surfactants self-assembled in 1D nanostructures
Source: RSC Adv. 2019 Oct 16;9(57):33071–9. doi: 10.1039/c9ra06390k (PMC9073239; doi:10.1039/c9ra06390k)
Supplement: RA-009-C9RA06390K-s001 [file RA-009-C9RA06390K-s001.pdf]

Electronic Supplementary Material (ESI) for RSC Advances.
This journal is © The Royal Society of Chemistry 2019

Monolayer formed by L-Asp Based Gemini Surfactants Self-Assembled in 1D Nanostructures

Borislav Anchev[1], Daniela S. Tsekova[1]*, Kristina M. Mircheva[2], Nikolay A. Grozev[2].

[1] Dept. of Organic Chemistry University of Chemical Technology and Metallurgy, Sofia 1756, Bulgaria; [2] Dept. of Physical Chemistry, Faculty of Chemistry and Pharmacy, Sofia University,,St. Kl. Ohridski", Sofia 1164, Bulgaria.
Tel: +359876589755 ; E-mail: d_tsekova@uctm.edu

Electronic Supporting Information

-Experimental methodology. p.2
-Synthetic details
-Characterization of new compounds.
- Reagents used p.6
- Additional data for the π-A isotherms (Fig. ESI-1.) p.6
- Data from conductometric determination of CAC (Fig. ESI-2.A-2.C.) p.7
- **log CAC** vs. ***n*** - number of carbon atoms in both alyphatic chains (Fig. ESI-3.) p.8
- Enlarged AFM images of LB films, presented also in the main paper (Fig. 4-6.) p.9

Chemical synthesis and purification

Synthesis of the precursor: N,N'-Bis-(Boc-L-Aspartyl)-1,6-diaminohexane (refered as Boc-A6)

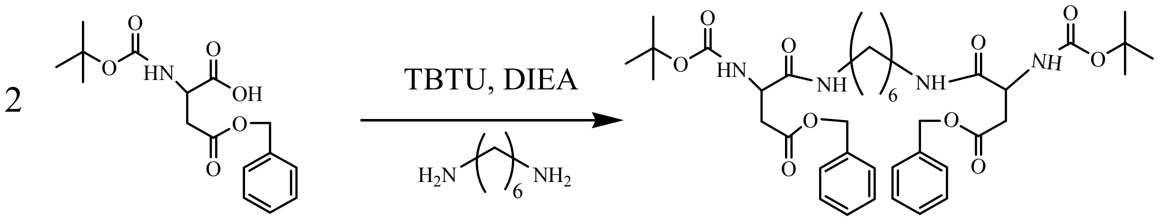

Boc-Asp(OBzl)-OH (10 g, 0.031 mol) together with of hexamethylenediamine (1.77 g, 0.015 mol) were dissolved in 20 mL dimethylformamide (DMF) at rooom temperature and cooled at 0 ºC. Then TBTU (10 g, 0.031 mol) was added, and finally N-diisopropylethylamine (DIPEA) (4.12 g, 5.6 mL, 0.032 mol) was dropped. The mixture was stirred overnight at room temperature. Afterwards, 8% $NaHCO_3$ was added until white precipitate formed. This mixture was left in the refrigator at 4-6 °C for 1-2 hours and the precipitation was filtered off and washed firstly with 8% $NaHCO_3$, then with 10% citric acid and finally with cool destilled water until neutral pH of the washing water. The solid crude was recrystallized from ethylacetate/hexane. The product was obtained after filtration as white crystals.

White solid (85% yield). M.p. 129 °C. ^{1}H NMR (600 MHz, $CDCl_3$) δ = 7.40 – 7.33 (m, 10H), 6.55 (t, *J* = 5.7 Hz, 2H), 5.75 (d, *J* = 7.5 Hz, 2H), 5.15 (q, *J* = 12.3 Hz, 4H), 4.52 (s, 2H), 3.28 (dt, *J* = 13.3, 6.7 Hz, 2H), 3.05 (dd, *J* = 17.1, 4.6 Hz, 2H), 2.75 (dd, *J* = 17.1, 6.5 Hz, 2H), 1.47 (s, 22H), 1.32 (s, 4H) ppm. ^{13}C NMR (151 MHz, $CDCl_3$) δ = 171.79, 170.59, 155.64, 135.41, 128.62, 128.40, 128.25, 80.55, 66.80, 50.66, 39.19, 36.16, 29.22, 28.31, 25.98 ppm. IR: ν = 3287.5; 2920.4; 2851.4; 1731.3; 1636.3; 1559,9; 1541.3; 1172.2 cm^{-1}. MS (ES) (m/z): Calcd. for $C_{38}H_{54}N_4O_{10}$ 726.38; found for $[M + H^+]$ 727.39.

Synthesis of 1a-d was done in two steps by deprotection of Boc-A6 and in situ acylation.

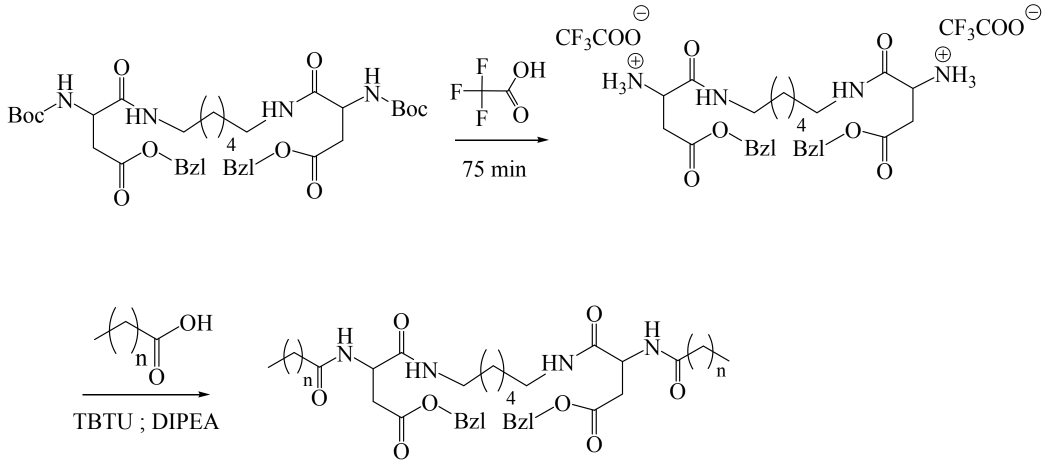

n = 0, 4, 8, 12

Mixture of Boc-A6 (1 eq.) and Trifluoroacetic acid (TFA) (24 eq.) was stirred about 75 minutes. The excess of TFA was removed by vacuum evaporation. The solid obtained was dissolved in min DMF (1g solid in 5-10 ml DMF) and DIEA was dropped in it so that pH 7 was achieved. This solution was cooled at an ice-bath and 2.4 eq. of the corresponding fatty acid, TBTU (2.4 eq.) and DIEA (2.4 eq.) were added successively to the cooled mixture. The reaction was left for 2 hours at 0°C and stirred 24 hours at room temperature. Then 8% $NaHCO_3$ was added to the reaction mixture, so that the product as white precipitate was observed. The flask was left at room temperature for 1 hour and then moved in the refrigerator at 4-6 °C for 1-2 hours where the precipitate consolidates. Afterwards the precipitation was filtered off and washed firstly with 8% $NaHCO_3$, then with 10% citric acid and finally with cool distilled water until neutral pH of the washing water. The product crude was recrystallized from ethylacetate/ petroleum ether and dried. All products were obtained after filtration as chromatographically pure white solids. The following compounds have been obtained according this procedure:

Compound ***1a***: White solid (95 % yield). M.p.78-79 °C. ^1H NMR (600 MHz, $CDCl_3$): δ = 7.45 – 7.31 (m, 10H), 7.10 (d, *J* = 8.4 Hz, 2H), 6.69 (t, *J* = 6.0 Hz, 2H), 5.32 – 5.06 (m, 4H), 4.94 – 4.78 (m, 2H), 3.32 (dq, *J* = 13.6, 6.8 Hz, 2H), 3.19 – 3.08 (m, 2H), 2.96 (dd, *J* = 16.9, 4.7 Hz, 2H), 2.75 (dd, *J* = 16.9, 7.1 Hz, 2H), 2.01 (s, 6H), 1.48 (t, *J* = 6.6 Hz, 4H), 1.29 (dd, *J* = 14.9, 7.1 Hz, 4H) ppm. ^{13}C NMR (151 MHz, $CDCl_3$): δ = 171.76, 170.53, 170.41, 135.35, 128.64, 128.46, 128.26, 66.88, 49.35, 38.57, 35.84, 28.99, 25.23, 23.14 ppm. IR: ν = 3289.4, 3067.6, 2933.4, 1731.3, 1641.9, 1541.3 cm^{-1}. MS (ES) (m/z): Calculated for $C_{32}H_{42}N_4O_8$ 610.30; found for [M + H^+] 611.32.

.

Compound ***1b***: White solid (55 % yield). M.p. 78-79 °C. ^1H NMR (600 MHz, $CDCl_3$): δ = 7.41 – 7.32 (m, 10H), 7.00 (d, *J* = 8.2 Hz, 2H), 6.68 (t, *J* = 5.9 Hz, 2H), 5.19 – 5.11 (m, 4H), 4.87 – 4.81 (m, 2H), 3.29 (dq, *J* = 13.4, 6.8 Hz, 2H), 3.18 – 3.11 (m, 2H), 2.96 (dd, *J* = 16.9, 4.6 Hz, 2H), 2.73 (dd, *J* = 16.9, 7.0 Hz, 2H), 2.24 – 2.18 (m, 4H), 1.64 (d, *J* = 6.7 Hz, 4H), 1.50 – 1.44 (m, 4H), 1.30

(ddd, J = 14.6, 10.5, 4.9 Hz, 12H), 0.90 (t, J = 7.1 Hz, 6H) ppm. ^{13}C NMR (151 MHz, $CDCl_3$): δ = 173.59, 171.87, 170.44, 135.37, 128.63, 128.44, 128.26, 66.86, 49.19, 38.81, 36.43, 35.77, 31.39, 29.08, 25.56, 25.25, 22.39, 13.96 ppm. IR: ν = 3287.5, 2927.8, 2858.9, 1731.3, 1634.4, 1559.5, 1539.4 cm^{-1}. MS (ES) (m/z): Calculated for $C_{40}H_{58}N_4O_8$ 722,43; found [M + H$^+$] 721.35. .

Compound ***1c***: White solid (90 % yield). M.p. 105-107 °C. ^{1}H NMR (600 MHz, $CDCl_3$): δ = 7.35 – 7.31 (m, 10H), 6.99 (d, J = 8.2 Hz, 2H), 6.68 (t, J = 5.8 Hz, 2H), 5.13 (d, J = 3.5 Hz, 4H), 4.82 (td, J = 7.2, 4.7 Hz, 2H), 3.27 (dq, J = 13.5, 6.8 Hz, 2H), 3.13 (dt, J = 12.4, 6.6 Hz, 2H), 2.96 – 2.91 (m, 2H), 2.71 (dd, J = 16.8, 7.0 Hz, 2H), 2.21 – 2.17 (m, 4H), 1.62 – 1.56 (m, 4H), 1.48 – 1.42 (m, 4H), 1.28 – 1.24 (m, 28H), 0.87 (t, J = 7.1 Hz, 6H) ppm. ^{13}C NMR (151 MHz, $CDCl_3$): δ = 173.61, 171.84, 170.46, 135.37, 128.63, 128.43, 128.26, 66.86, 49.20, 38.80, 36.48, 35.81, 31.87, 29.47, 29.36, 29.29, 29.27, 29.07, 25.59, 25.54, 22.68, 14.14 ppm. IR: ν = 3297.5, 2927.8, 2853.3, 1727.6, 1634.4, 1559.5, 1541.3 cm^{-1}.

Compound ***1d***: White solid (95 % yield). M.p. 130 °C. ^{1}H NMR (600 MHz, $CDCl_3$): δ = 7.40 – 7.32 (m, 10H), 7.01 (d, J = 8.3 Hz, 2H), 6.70 (t, J = 5.9 Hz, 2H), 5.21 – 5.08 (m, 4H), 4.88 – 4.79 (m, 2H), 3.29 (dq, J = 13.4, 6.8 Hz, 2H), 3.15 (td, J = 12.2, 6.4 Hz, 2H), 2.96 (dd, J = 16.8, 4.7 Hz, 2H), 2.73 (dd, J = 16.8, 7.0 Hz, 2H), 2.26 – 2.15 (m, 4H), 1.66 – 1.55 (m, 4H), 1.47 (t, J = 7.0 Hz, 4H), 1.27 (d, J = 10.9 Hz, 44H), 0.90 (t, J = 7.0 Hz, 6H) ppm. ^{13}C NMR (151 MHz, $CDCl_3$): δ = 173.61, 171.84, 170.46, 135.37, 128.63, 128.43, 128.26, 66.86, 49.20, 38.79, 36.48, 35.81, 31.93, 29.70, 29.67, 29.65, 29.53, 29.38, 29.29, 29.07, 25.59, 25.53, 22.71, 14.15 ppm. IR: ν = 3297.0, 2917.3, 2849.5, 1703.3, 1640.6, 1588.3, 1542 cm^{-1}. MS (ES) (m/z): Calculated for $C_{56}H_{90}N_4O_8$ 946,68; found [M + H$^+$] 947.69. .

Synthesis of 2a-d by hydrogenolysis of the benzyl groups of 1a-d

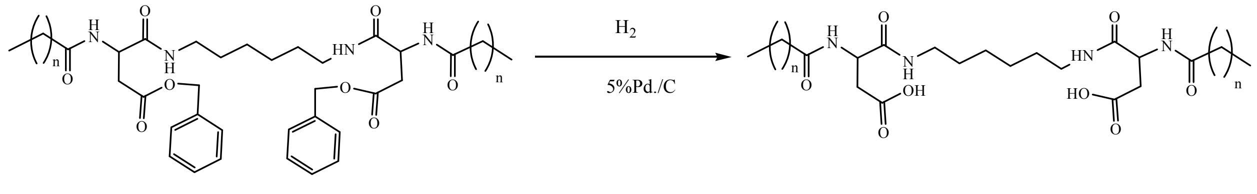

1 a-d 2 a-d

Compound 1 (1 g) was dissolved in 150 ml methanol and placed in a 250 ml round bottom flask. Then 5 % Pd/C (200 mg) was added to the above mixture. The flask was connected to a hydrogen storage bottle by means of a reducer. Hydrogen was bubbled through the solution stirred on a magnetic stirrer for about 8-10 hours. The reaction course has been followed by a TLC, using solvent system 9:1 chloroform: acetic acid, until the benzyl ester spot vanished. The catalyst was

filtered at the end of reaction and washed by small portions (about 5 ml) of methanol. After removing of methanol from the filtrate, pure product was obtained. Compounds synthesised in this manner are as follow:

Compound ***2a***: White solid (90 % yield). M.p. 159-160 °C. ^{1}H NMR (600 MHz, DMSO): δ = 8.10 (d, *J* = 8.1 Hz, 1H), 7.82 (t, *J* = 5.7 Hz, 1H), 4.60 – 4.38 (m, 1H), 3.07 – 2.93 (m, 2H), 2.58 (dt, *J* = 31.4, 15.7 Hz, 1H), 2.48 – 2.35 (m, 1H), 1.82 (s, 3H), 1.34 (d, *J* = 6.7 Hz, 2H), 1.20 (s, 2H) ppm. ^{13}C NMR (151 MHz, DMSO): δ = 172.30, 170.83, 169.68, 49.95, 38.98, 36.95, 29.37, 26.36, 23.06 ppm. IR: ν = 3285.6: 2920.4; 2851.4; 1701.5; 1636.3; 1541.3; 1287.8; 1183.4; 1112.6 cm^{-1}. MS (ES) (m/z): Calculated for $C_{18}H_{30}N_4O_8$ 430,21; found [M + H$^+$] 431.21. .

Compound ***2b***: White solid (70 % yield). M.p.135-136 °C. ^{1}H NMR (600 MHz, DMSO): δ = 8.04 (d, *J* = 8.1 Hz, 1H), 7.76 (t, *J* = 5.7 Hz, 1H), 4.50 (dd, *J* = 14.1, 7.9 Hz, 1H), 3.00 (dd, *J* = 12.9, 6.7 Hz, 2H), 2.60 (dd, *J* = 16.2, 6.1 Hz, 1H), 2.42 (dd, *J* = 16.2, 7.8 Hz, 1H), 2.08 (t, *J* = 7.5 Hz, 2H), 1.51 – 1.43 (m, 2H), 1.34 (s, 2H), 1.25 (dd, *J* = 14.7, 7.5 Hz, 2H), 1.19 (dd, *J* = 10.0, 5.2 Hz, 4H), 0.84 (t, *J* = 7.2 Hz, 3H) ppm. ^{13}C NMR (151 MHz, DMSO): δ = 172.67, 172.31, 170.87, 49.84, 38.94, 36.92, 35.59, 31.24, 29.38, 26.34, 25.30, 22.36, 14.34 ppm. IR: ν = 3287.5; 2918.5; 2851.4; 1701.5; 1634.4; 1541.3; 1291.5; 1224.4; 1120.1 cm^{-1}. MS (ES) (m/z): Calculated for $C_{26}H_{46}N_4O_8$ 542,33; found [M + H$^+$] 543.34.

Compound ***2c***: White solid (90 % yield). M.p. 144-145 °C. ^{1}H NMR (600 MHz, DMSO): δ = 8.03 (d, *J* = 8.2 Hz, 1H), 7.74 (t, *J* = 5.7 Hz, 1H), 4.50 (dd, *J* = 14.2, 7.9 Hz, 1H), 3.00 (dd, *J* = 13.2, 6.7 Hz, 2H), 2.61 (dd, *J* = 16.2, 6.2 Hz, 1H), 2.42 (dd, *J* = 16.2, 7.8 Hz, 1H), 2.07 (t, *J* = 7.4 Hz, 2H), 1.48 – 1.43 (m, 2H), 1.34 (s, 2H), 1.22 (s, 14H), 0.85 (t, *J* = 7.0 Hz, 3H) ppm. ^{13}C NMR (151 MHz, DMSO): δ = 172.66, 172.28, 170.86, 49.81, 38.95, 36.84, 35.63, 31.77, 29.39, 29.39, 29.32, 29.17, 29.05, 26.36, 25.63, 22.58, 14.44 ppm. IR: ν = 3285.6; 2918.5; 2851.4; 1701.5; 1634.4; 1541.3; 1291.5; 1224.4; 1120.1 cm^{-1}. MS (ES) (m/z): Calculated for $C_{34}H_{62}N_4O_8$ 654,46; found [M + H$^+$] 654.46.

Compound ***2d***: White solid (55 % yield). M.p. 137-138 °C. ^{1}H NMR (600 MHz, DMSO): δ = 8.03 (d, *J* = 7.7 Hz, 1H), 7.74 (t, *J* = 5.8 Hz, 1H), 4.50 (dd, *J* = 14.6, 7.8 Hz, 1H), 3.13 – 2.97 (m, 2H), 2.60 (dd, *J* = 16.2, 6.0 Hz, 1H), 2.41 (dd, *J* = 16.2, 7.7 Hz, 1H), 2.07 (t, *J* = 7.3 Hz, 2H), 1.45 (s, 2H), 1.34 (s, 2H), 1.23 (s, 22H), 0.85 (t, *J* = 6.9 Hz, 3H) ppm. ^{13}C NMR (151 MHz, DMSO): δ = 172.63, 172.31, 170.8, 49.83, 38.94, 36.88, 35.62, 31.77, 29.55, 29.52, 29.50, 29.44, 29.39, 29.39, 29.32, 29.20, 29.06, 26.34, 25.63, 22.58, 14.44 ppm. IR: ν = 3291,2; 29916.6; 2851.4; 1701.5; 1636.3; 1541.5, 1291.5 1121.9; 1120.1 cm^{-1}. MS (ES) (m/z): Calculated for $C_{42}H_{78}N_4O_8$ 766,58; found [M + H$^+$] 767.59.

General procedure for obtaining of double sodium salts of compounds 2a-d

Compound ***2*** (1g) was mixed with 2 ml distilled water and stirred on a magnetic stirrer for two hours at room temperature. Equivalently to the used acid (compound ***2***) NaOH 1M solution was

added to the above mixture. Acid was dissolving parallel to its double sodium salt formation and solution pH was changing from alkaline to neutral adequately. Then water was evaporated and the corresponding salt was obtained. The yields were quantitative 98-100%.

Reagents used

Boc-Asp(OBzl)-OH (Alfa Aesar); Amphiphilic fatty acids, chlorides and anhydrides (Alfa Aesar); TBTU (Iris Biotech GmbH); DIEA [*N*,*N*-Diisopropylethylamine] (Alfa Aesar), DMF [Dimethylformamide] (Merck); Ethylacetate (Merck); Hexane (Merck); $NaHCO_3$ (Merck); Trifluoroacetic acid (Alfa Aesar); Citric acid (Merck); Chloroform (Merck); Methanol (Merck); 5 % Pd/C (Sigma-Aldrich).

Additional data for the π-A isotherms

For a short time interval (e.g several weeks) the isotherms are very reproducible (Fig. 1S, isotherm (1))., but a few months later a shift to bigger molecular areas was observed (see Fig. 1S, isotherm (2)). This indicates that some reorganization processes take place, perhaps some aggregates presented at first study became fully or partially dissolved during these time.

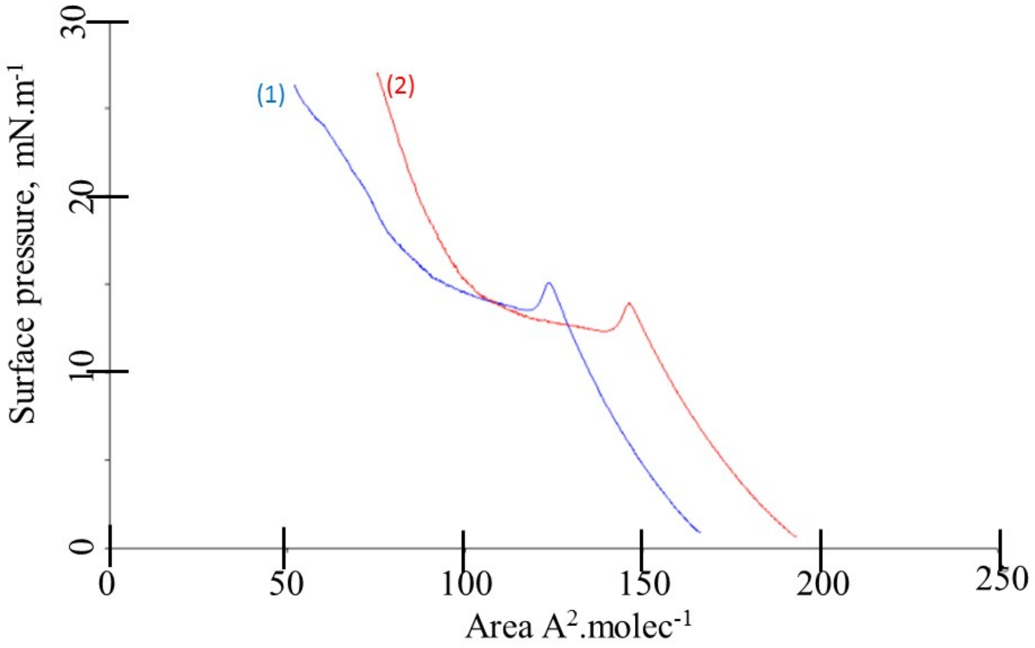

Fig. ESI-1. Two adsorption isotherm of **2d** obtained from one and the same sample in 6 months period. Blue (1) – fresh solution; red (2) - 6 months later.

Data from conductometric determination of CAC

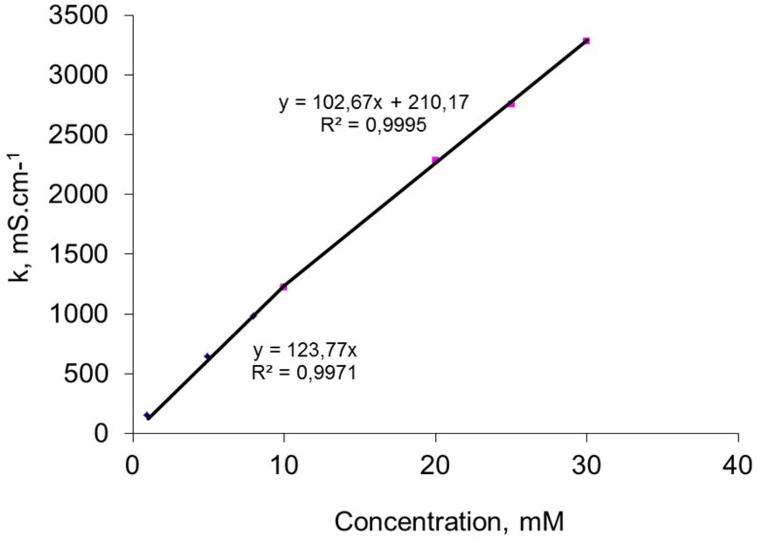

Fig. ESI-2A. Specific conductivity of sodium salts of **2b** solutions. CAC is 10 mM. The error of each value in the graphic is ≤ 20%.

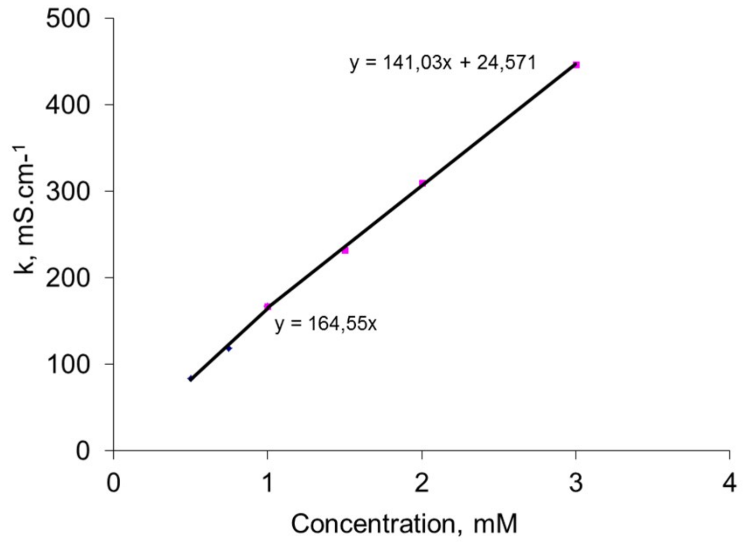

Fig. ESI-2B. Specific conductivity of sodium salts of **2c** solutions. CAC is 1 mM. The error of each value in the graphic is ≤ 20%.

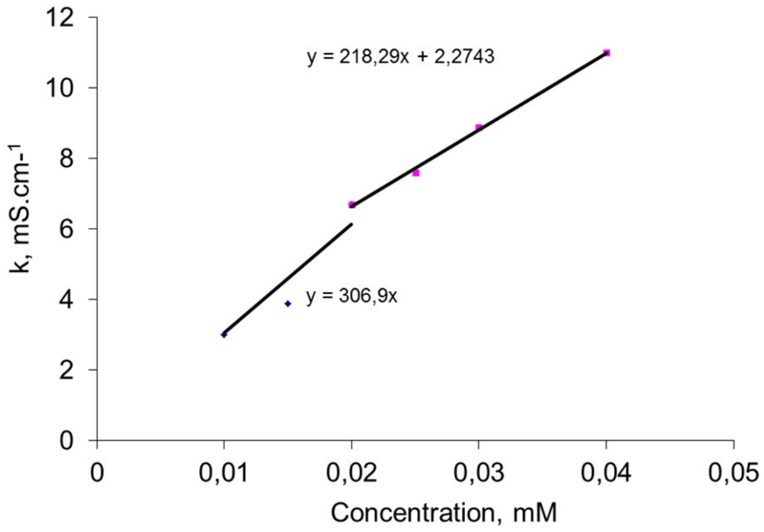

Fig. ESI-2C. Specific conductivity of sodium salts of **2d** solutions. CAC is 0.02 mM. The error of each value in the graphic is ≤ 20%.

The relationship of log CAC vs. carbon atoms in the hydrophobic chains is presented in Fig. 3S and shows close to linear dependence, which is expected for the surfactants molecules.

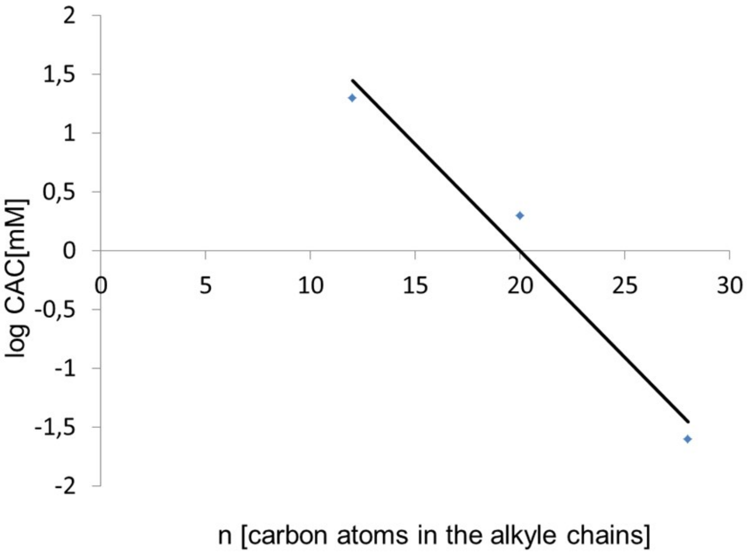

Fig. ESI-3. log CAC vs. ***n*** - number of carbon atoms in both alyphatic chains linked to the N-sides of the surfactants: ***n*** = 12 (compound **2b**), 20 (compound **2c**) and 28 (compound **2d**).

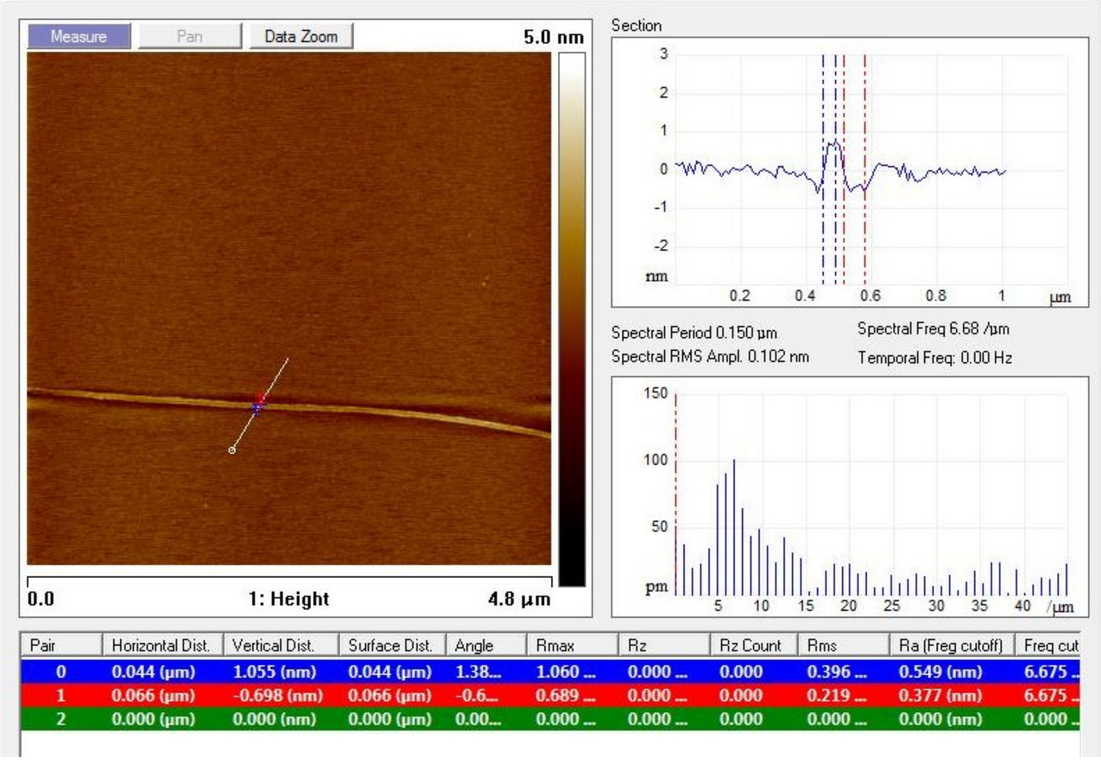

Fig. ESI-4A. AFM image of a sample (on coated mica substrate) of the compound **1c** before the collapse at $\pi = 15$ mN.m^{-1}; (This is enlarged image of the Fig.4.C. in the main paper).

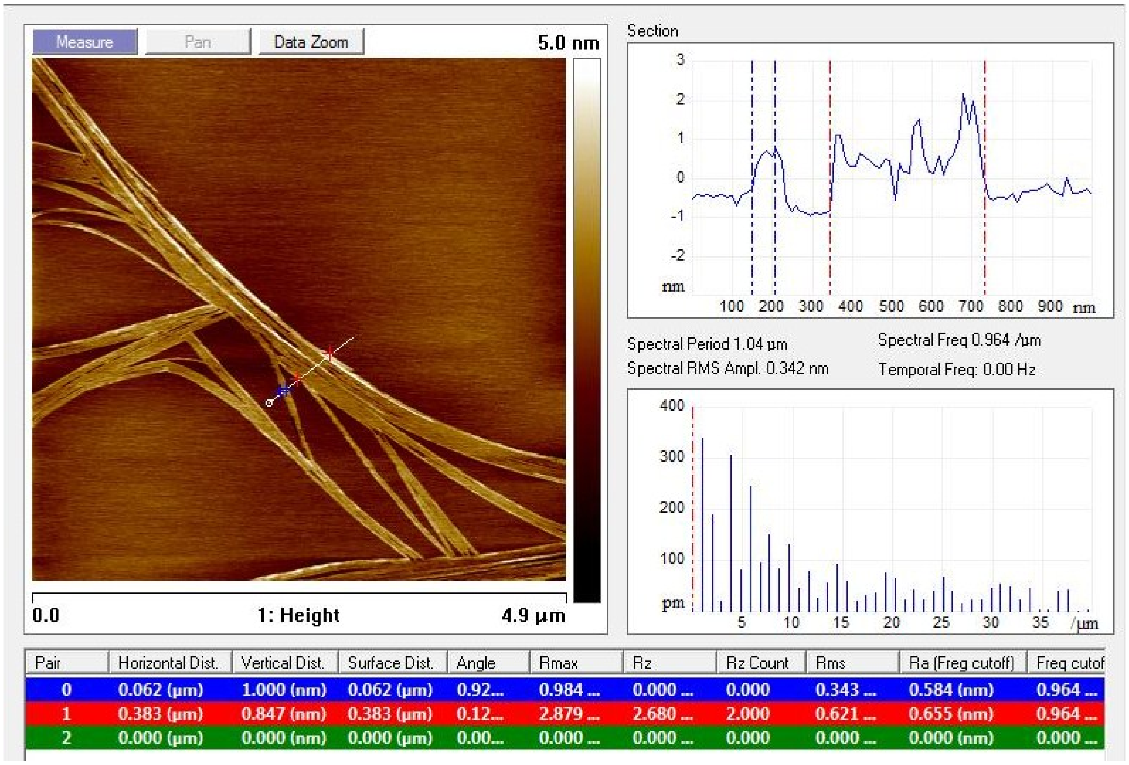

Fig. ESI-4B. AFM image of a sample (on coated mica substrate) of the compound **1c** after the collapse at $\pi = 22$ mN.m^{-1}; (This is enlarged image of the Fig.4.F. in the main paper).

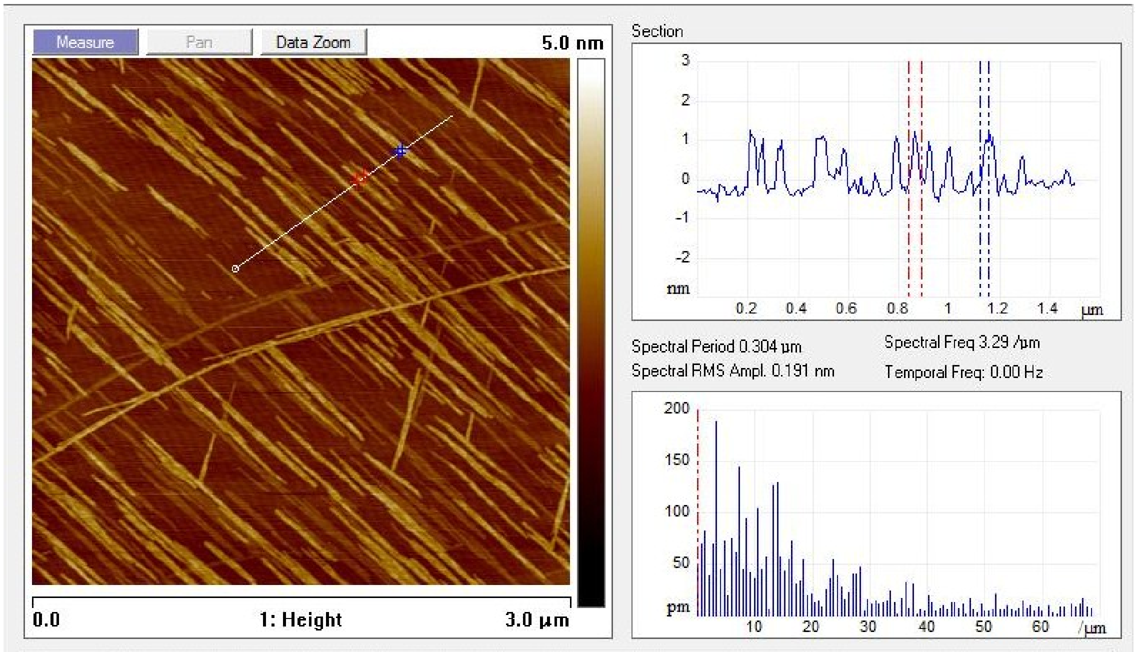

Pair	Horizontal Dist.	Vertical Dist.	Surface Dist.	Angle	Rmax	Rz	Rz Count	Rms	Ra (Freq cutoff)	Freq cut
0	0.029 (µm)	1.168 (nm)	0.029 (µm)	2.31...	1.208 ...	0.000 ...	0.000	0.447 ...	0.665 (nm)	3.289 ...
1	0.051 (µm)	0.165 (nm)	0.051 (µm)	0.18...	1.293 ...	0.000 ...	0.000	0.435 ...	0.477 (nm)	3.289 ...
2	0.000 (µm)	0.000 (nm)	0.000 (µm)	0.00...	0.000 ...	0.000 ...	0.000	0.000 ...	0.000 (nm)	0.000 ...

Fig. ESI-5A. AFM image of a sample (on coated mica substrate) of the compound **1d** before the collapse at $\pi = 13$ mN.m^{-1}; (This is enlarged image of the Fig.5.B. in the main paper).

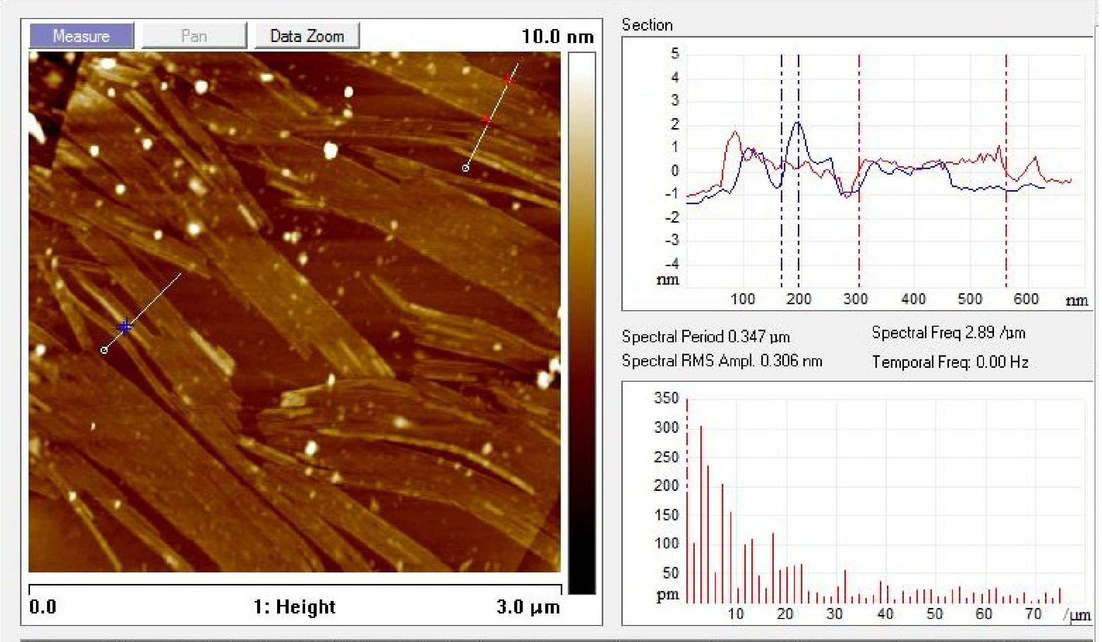

Pair	Horizontal Dist.	Vertical Dist.	Surface Dist.	Angle	Rmax	Rz	Rz Count	Rms	Ra (Freq cutoff)	Freq cuto
0	0.033 (µm)	2.766 (nm)	0.033 (µm)	4.82...	2.785 ...	0.000 ...	0.000	1.078 ...	1.332 (nm)	1.525 ...
1	0.262 (µm)	0.094 (nm)	0.262 (µm)	0.02...	1.174 ...	1.174 ...	2.000	0.212 ...	0.265 (nm)	2.886 ...
2	0.000 (µm)	0.000 (nm)	0.000 (µm)	0.00...	0.000 ...	0.000 ...	0.000	0.000 ...	0.000 (nm)	0.000 ...

Fig. ESI-5B. AFM image of a sample (on coated mica substrate) of the compound **1d** after the collapse at $\pi = 27$ mN.m^{-1}; (This is enlarged image of the Fig.5.D. in the main paper).

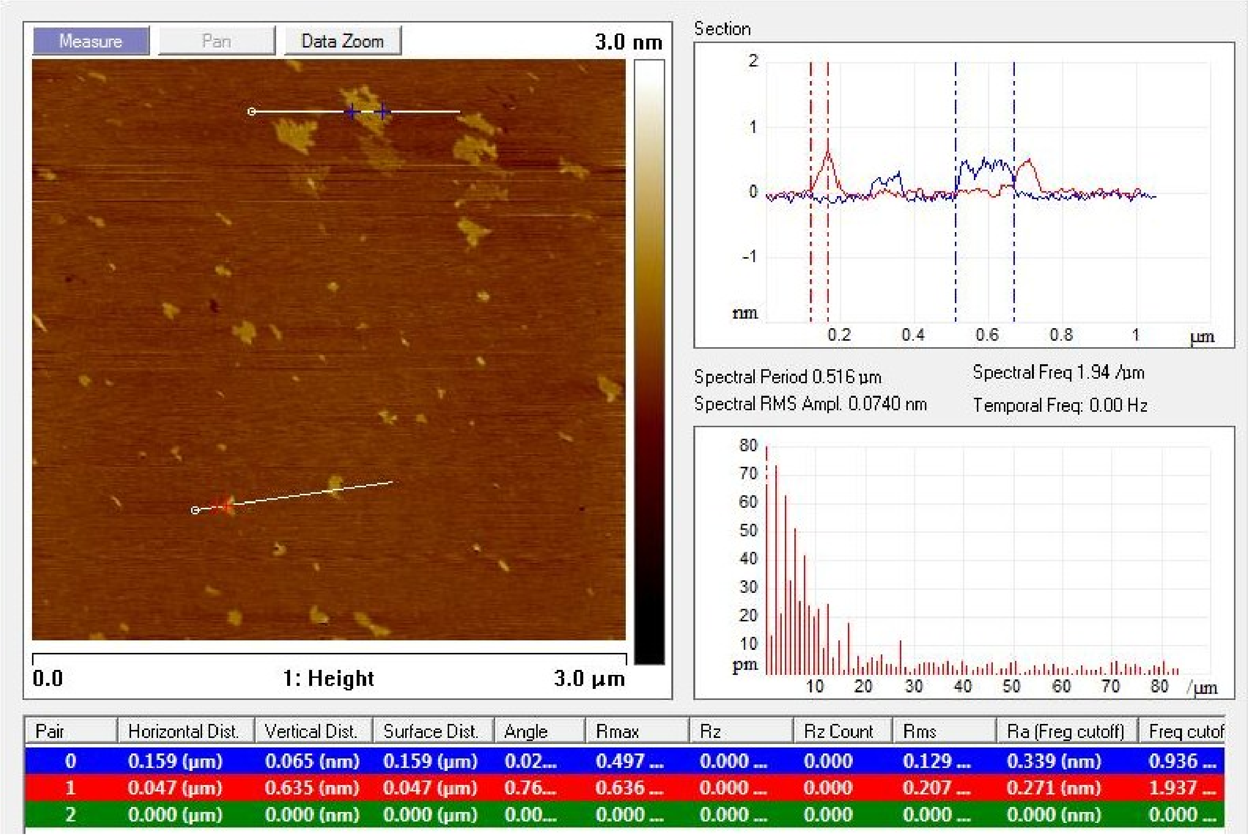

Fig. ESI-6. AFM image of a sample (on coated mica substrate) of the compound **2d** in a monolayer (This is enlarged AFM image of the Fig.6. in the main paper).
